# Effects of the linoleic acid/docosahexaenoic acid ratio and concentration inducing autophagy in Raw264.7 cells against *Staphylococcus aureus*

**DOI:** 10.3164/jcbn.19-95

**Published:** 2020-04-17

**Authors:** Li-Ying Xu, Min Mu, Man-Li Wang, Jin-Cheng Liu, Yuan-Jie Zhou, Jing Wu, Bing-You Jiang, Ming-Gong Chen, Dong Hu, Xing-Rong Tao

**Affiliations:** 1 School of Medicine, Department of Medical Frontier Experimental Center, Anhui University of Science and Technology, 168 Taifeng Road, Huainan City, Anhui Province 232001, China; 2 Key Laboratory of Industrial Dust Control and Occupational Health of the Ministry of Education of Anhui Higher Education Institutes, 168 Taifeng Road, Huainan City, Anhui Province 232001, China; 3 Key Laboratory of Industrial Dust Deep Reduction and Occupational Health and Safety of Anhui Higher Education Institutes, 168 Taifeng Road, Huainan City, Anhui Province 232001, China

**Keywords:** *n*-6/*n*-3 polyunsaturated fatty acids (PUFAs), autophagy, *Staphylococcus aureus*, concentration, ratio

## Abstract

Our study was to understand the autophagy induce by different ratios and concentrations of LA/DHA on Raw264.7 cell, and then to investigate the effect of Raw264.7 autophagy on the clearance of *Staphylococcus aureus*. Raw264.7 cells was treated by LA/DHA in different concentrations (50/100 µmol/L) and ratios (4:1, 6:1, 8:1, 1:4, 1:6 and 1:8) for 6/12/24 h, cell viability assay was assessed by Cell Counting Kit-8, LC3B, p62, P-mTOR, P-Akt, P-PI3K and BECN 1 were detected by the Western blot. LA/DHA could induce autophagy of Raw264.7 cells through the PI3K-Akt-mTOR signaling pathway, the strong effect on autophagy by the concentration is 100 µmol/L, the ratio is 6:1 of LA/DHA, and the treatment time is 24 h. Compared with the images in the control group obtained by merging red and green fluorescence channels, the treatment of LA, DHA in a ratio of 6:1 at a concentration of 100 µmol/L for 24 h significantly lead to a substantial number of autophagosomes (yellow) as well as autolysosomes (red), enhancing autophagy flux. Autophagy induce by LA/DHA can devour and damage intracellular and extracellular *Staphylococcus aureus*. These results indicate that LA/DHA cloud induce autophagy and enhance the phagocytosis and killing ability of macrophages to intracellular parasitic bacteria.

## Introduction

Autophagy is a process in which cells use lysosomes to degrade their damaged organelles and macromolecular substances.^(1)^ Essentially, it is a self-engulfing of the cell.^(2)^ Autophagy can be seen in both physiological and pathological processes of the body.^(3)^ Some diseases like cardiovascular diseases, tumors and infectious diseases may caused by autophagy disorders.^(4,5)^ Thus, in the treatment of these diseases, the strategy of up-regulation or inhibition of autophagy is also worthy of our attention.

Accumulating evidences have been shown that *n*-6/*n*-3 polyunsaturated fatty acids (PUFAs) can not only induce cell autophagy but also affect the ability of host to resist intracellular pathogen autophagy.^(6,7)^ Furthermore, related genes and signaling pathways have also been detected.^(7)^ While the most interesting finding is that docosahexaenoic acid (DHA) and eicosapentaenoic acid (EPA) in *n*-3 PUFAs can enhance the expression of protein light chain 3 (LC3) to treat lung cancer.^(8)^ At present, most of the literature indicate that DHA can induce cell apoptosis, but few scholars have pay attention to the role of DHA in inducing autophagy.^(9,10)^ Experimental studies shows that DHA increases the number of protein LC3 and autophagosome by regulating the levels of microtubule-associated proteins, at the same time, the effect of other autophagosomes has not been weakened, in short, DHA can induce autophagy.^(9)^ Moreover, *n*-6 PUFAs as for linolenic acid (LA) and dihomo-γ-linolenic acid (DGLA) also could induce autophagy independently through mTOR pathway.^(10)^

In recent years, due to the proportion of the consumption of vegetable oil intake in China has been increasing, the health problems caused by it cannot be ignored. There are a lot of misconceptions about the correct supplement of *n*-6/*n*-3 PUFAs in the world, especially there is still a debate about the reasonable ratio of the *n*-6/*n*-3 PUFAs.^(11)^ Accumulating evidences have demonstrated the high ratio of *n*-6/*n*-3 PUFAs in diet is closely related to the high incidence of atherosclerosis, diabetes, breast cancer and other diseases.^(12–15)^ So the ratio and number of *n*-6/*n*-3 PUFAs in the dietary intake are particularly important. However, in recent years, the dietary pattern of Chinese has been gradually westernized, and the *n*-6/*n*-3 PUFAs ratio has gradually increased, and some areas have reached or even exceeded the ratio of *n*-6/*n*-3 PUFAs in the American diet.

*Staphylococcus aureus* is a clinically common pathogen. As a facultative intracellular pathogen, it has been shown to invade and replicate in phagocytic and non-phagocytic cells,^(16–18)^ causing pustules, cellulitis, food poisoning, toxic shock syndrome, necrotizing pneumonia, endocarditis and other diseases. Bravo-Santano *et al.*^(19)^ and Wang *et al.*^(20)^ proposed that *Staphylococcus aureus* can activate autophagy after invading host cells. Further research has shown that the PI3K/Akt-Beclin1 signaling pathway positively regulates phagocytosis in *Staphylococcus aureus*-infected macrophages.^(21)^ At present, there are still few studies on LA and DHA inducing autophagy in Raw264.7 cells, and the effect of this autophagy on *Staphylococcus aureus*. The optimal ratio and concentration of LA and DHA inducing autophagy and mechanism are not clear. Therefore, this study further elucidated the autophagy induced by different ratio and concentration of LA and DHA on Raw264.7 cell, and then to investigate the effect of Raw264.7 cell autophagy on the clearance of *Staphylococcus aureus*.

## Materials and Methods

### Chemicals

LA and DHA (Sigma, St. Louis, MO) was dissolved by anhydrous ethanol, add 0.1 N NaOH at 55°C, then dissolve LA and DHA in 10% BSA at molar ratio 1:1. The final concentration of LA and DHA was 14.70 µmol/ml.

### Cell and bacterium culture

Raw264.7 cells was grown in DMEM medium (Thermo Fisher Scientific, Waltham, MA) supplemented with 10% fetal calf serum (GIBCO, Grand Island, NY) at 37°C in a 5% CO_2_ atmosphere. To prevent cell differentiation, mRFP-GFP-LC3 cells were cultured in RPMI-1640 (Thermo Fisher Scientific) medium under the same environment.

*Staphylococcus aureus* CICC 21600 were cultured in the ordinary solid AGAR medium, washed with 0.9% sterile normal saline. Fresh *Staphylococcus aureus* suspension was obtained after elution and mixing. OD value of *Staphylococcus aureus* suspension was measured and adjusted to 0.08.

### Cell viability assay

Cell viability assay Cell Counting Kit-8 (Beyotime Biotechnology, Shanghai, China) was used to assess cell viability according to the manufacturer’s instructions.

### Western blot

Raw264.7 cells were resuspended and tiled to multiple six-well plates. After LA/DHA in different concentrations (50/100 µmol/L) and ratios (4:1, 6:1, 8:1, 1:4, 1:6 and 1:8) for 6/12/24 h, RIPA (Beyotime) containing 1% PMSF was added and the six-well plates were placed in a 4°C shaking bed for cracking overnight. After cell fragments were removed by centrifugation at a speed of 16,000 rpm for 10 min, collect protein and determine the protein content of the cellular lysates by using BCA protein concentration determination kit (Beyotime), 30 µg of the protein samples were loaded on SDS-PAGE gel and electrophoresed. Then transfer the electrophoresed proteins on to the Polyvinylidene difluoride (PVDF) membrane using wet transfer, blocking, adding anti-LC3B antibody (#ab192890; Abcam, Cambridge, UK, dilution-1:2,000), anti-P62 antibody (#18420-1-AP; Proteintech, Manchester, GM, dilution-1:1,000), anti-internal reference antibody (beta Actin, #20536-1-AP; Proteintech, dilution-1:10,000), anti-P-Akt antibody (#4060S; Cell Signaling Technology, Danvers, MA, dilution-1:2,000), anti-BECN1 antibody (#A7353; ABclonal, Wuhan,China, dilution-1:1,000), anti-P-mTOR antibody (#2971S; Cell Signaling Technology, dilution-1:1,000), anti-mTOR antibody (#2972S; Cell Signaling Technology, dilution-1:1,000) and anti-P-PI(3)K antibody (#4228S; Cell Signaling Technology, dilution-1:1,000) and finally incubated overnight in a shaking bed at 4°C. This was followed by incubation in dark for 40 min with anti-rabbit HRP antibody (#SA00001-2; Proteintech, dilution-1:10,000). Chemiluminescence was then used to detect the proteins on the blot in the gel imaging analysis system after treatment with ECL developer (Thermo Fisher Scientific). Save the image and quantitate protein expression levels using ImageJ software.

### Extracellular bacteria count

It was divided into control group, LA/DHA group (LA/DHA for 24 h at the concentration was 100 µmol/L, the ratio was 6:1), positive control group [Rapamycin, (RAPA)], Wortmanninan group, Wortmannin intervention group [LA/DHA (6:1, 100 µmol/L) + Wortmannin]. The Raw264.7 cells in five groups cultured at 37°C and 5% CO_2_ for 24 h. Washing with PBS after absorbing and discarding the culture medium, then add *Staphylococcus aureus* solution with OD600 value of 0.08 to the cells at a ratio of 10:1 MOI for 4 h. After 4-h phagocytosis, the supernatant and PBS used for twice cleaning were collected. After mixing, they were diluted by 10 times, inoculated onto the plate with PCA (plate count agar), next cultured overnight at 37°C for 24–48 h until the visible colonies were grown, and then counted with the colony counter.

### Intracellular bacteria count

Add *Staphylococcus aureus* solution with OD600 value of 0.08 to the cells at a ratio of 10:1 MOI for 4 h, absorb and discard the culture medium. After washing cells with PBS, culture medium containing 100 µg/ml gentamicin was added to kill the remaining undevoured extracellular bacteria for 1 h. LA/DHA (6:1, 100 µmol/L) were used to treat the Raw264.7 cells which had ingested the same number of *Staphylococcus aureus* for 24 h according to the grouping and treatment methods in Extracellular bacteria count. Washing cells with PBS again after absorbing and discarding the culture medium. Then 0.1% Triton X-100 was added to rupture the membrane for 20 min at room temperature. Blow and beat the cells and collect the liquid. After mixing, culture and count the number of colonies according to the method in Extracellular bacteria count (bacterial phagocytosis).

### Statistical analysis

Data were expressed as mean ± SE, Statistics was calculated using either unpaired student *t* test or One-way analysis of variance. SPSS 16.0 (SPSS, Inc., Chicago, IL) was used and *p*<0.05 was considered statistically significant. All graphs were produced using GraphPad Prism software. The dots marked by mRFP, GFP were analyzed using ImageJ software.

## Results

### The effects of 6, 12, 24 h and different ratio, concentration LA/DHA on autophagy in Raw264.7 cell

**Fig. 1:**
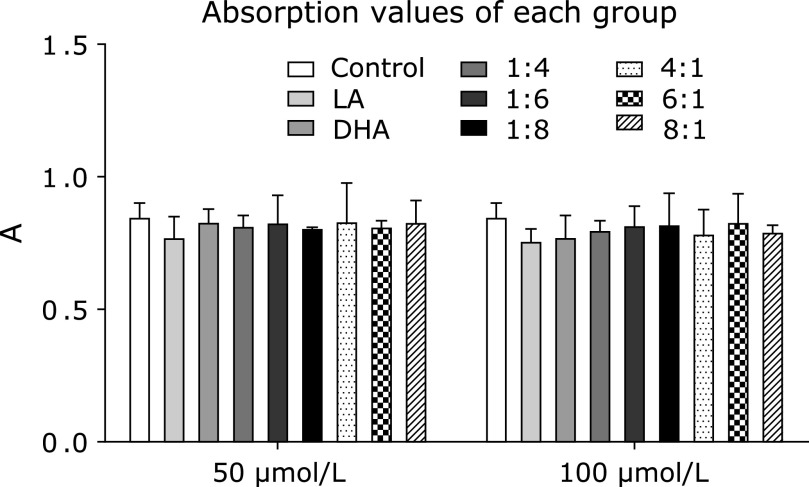
LA/DHA have no toxic effect on Raw264.7 cell proliferation activity. The results of Cell Counting Kit-8 assay exhibited that Raw264.7 cells showed no significant increase or decrease in proliferation activity after 24 h of action by LA and DHA in different proportions and concentrations, and the difference was not statistically significant (*p*>0.05).

 Compared with the control group, Cell Counting Kit-8 assay exhibited that after different ratio and concentration of LA/DHA treated with Raw264.7 cells, there was no significant change in cell viability, and the difference was not statistically significant. (*p*>0.05) (Fig. 1).

**Fig. 2:**
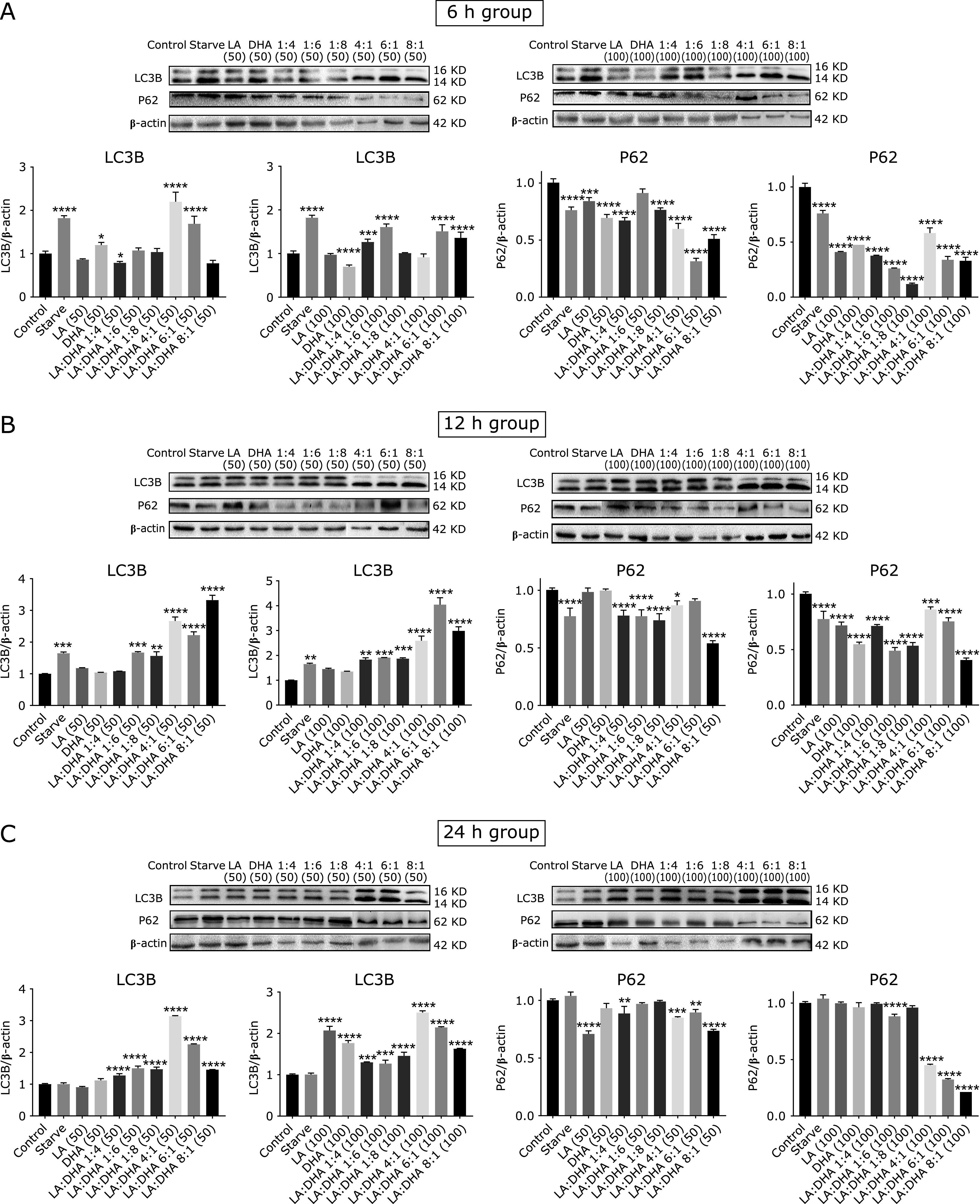
LA/DHA can induce autophagy. (A)–(C) respectively showed the expression levels of LC3B and P62 in Raw 264.7 cells after 6/12/24 h action of the LA and DHA. To elucidate how different concentration and ratio of LA/DHA affect the macrophage autophagy, we treated the Raw264.7 cells with different concentrations (50/100 µmol/L) and ratios (4:1, 6:1, 8:1,1:4, 1:6 and 1:8) LA/DHA for 6/12/24 h, and measured the levels of LC3B-I, LC3B-II, β-actin, and P62 by Western blot analysis. The results showed that the expression of LC3B in the LA/DHA group increased while the P62 decreased with the difference being statistically significant (*p<0.05, **p<0.01, ***p<0.001, ****p<0.0001).

To study the effects of different concentration and ratio of LA/DHA on autophagy, the levels of LC3B-I and LC3B-II were determined by Western blot analysis. The LC3 protein, a specific constituent of the autophagosomal membrane, is the only highly specific marker of the autophagosome presently. It is known to exist in two forms: LC3B-I (the 16 kDa cytosolic form) and LC3B-II [formed upon conjugation of LC3B-I to Phosphatidylethanolamine (PE) has molecular weight of 14 kDa]. LC3B-II is recruited to the inner as well as outer autophagosomal membranes.^(22)^ In Raw264.7 cell, the expression level of LC3B are significantly increased and P62 decreased in each LA/DHA group, the difference is statistically significant (*p*<0.05) (Fig. 2).

### mRFP-GFP-LC3 fluorescent spot

**Fig. 3:**
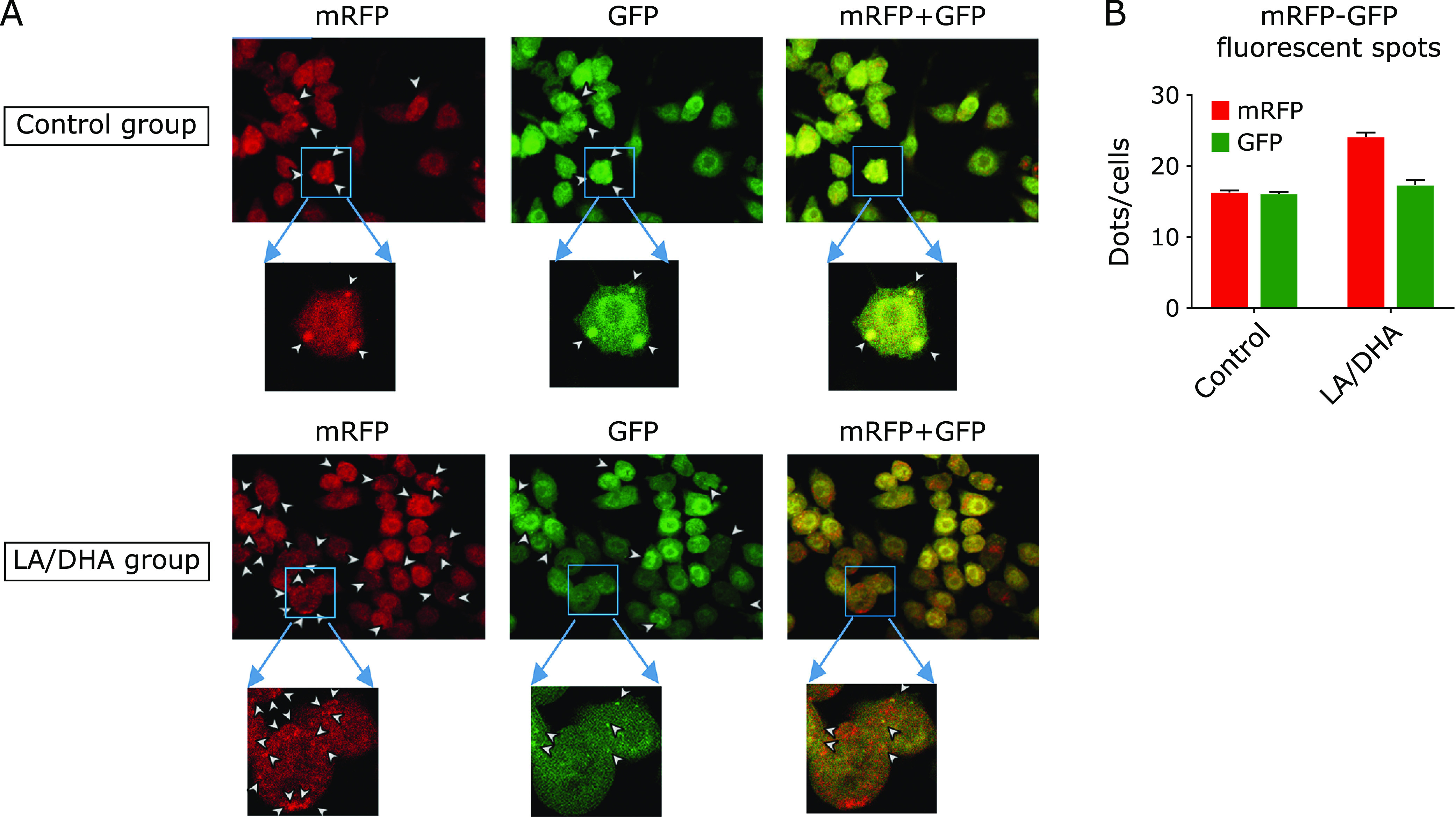
mRFP-GFP-LC3 fluorescent spot, LA, DHA enhance autophagy flux. As shown in the figure, green spots are acid-sensitive GFP proteins, red spots are autophagosomal lysosomal (mRFP), and yellow spots are autophagosomal (RFP + GFP). (A) respectively showed the overall and local enlarged images of mRFP-GFP-LC3 fluorescence spots under the action of LA/DHA with a concentration of 100 µmol/L and a ratio of 6:1 for 24 h. The analysis software ImageJ is used to analyze the level of red or green fluorescent spots in the image of each group, the bar graph was made and presented as (B). (B) showed the difference in the ratio of green and red fluorescence points between the control group and the LA/DHA group.

To confirm the ability of LA/DHA to induce autophagy, infected Raw264.7 cells were examined for puncta formation by confocal microscopy. mRFP-GFP-LC3 expressing cells were used for this assay. As shown in the A (Fig. 3), the control group presented yellow fluorescent spots after merging, and the LA/DHA group presented red and yellow fluorescent spots. The analysis software ImageJ is used to analyze the level of red or green fluorescent spots in the image of each group, the bar graph was made and presented as Fig. 3B. Compared with the control group, the treatment of LA, DHA in a ratio of 6:1 at a concentration of 100 µmol/L significantly lead to a substantial number of autophagosomes (yellow) as well as autolysosomes (red), enhancing autophagy flux and the autophagy flux was smooth.

### LA/DHA induce autophagy depends on mTOR activation

**Fig. 4:**
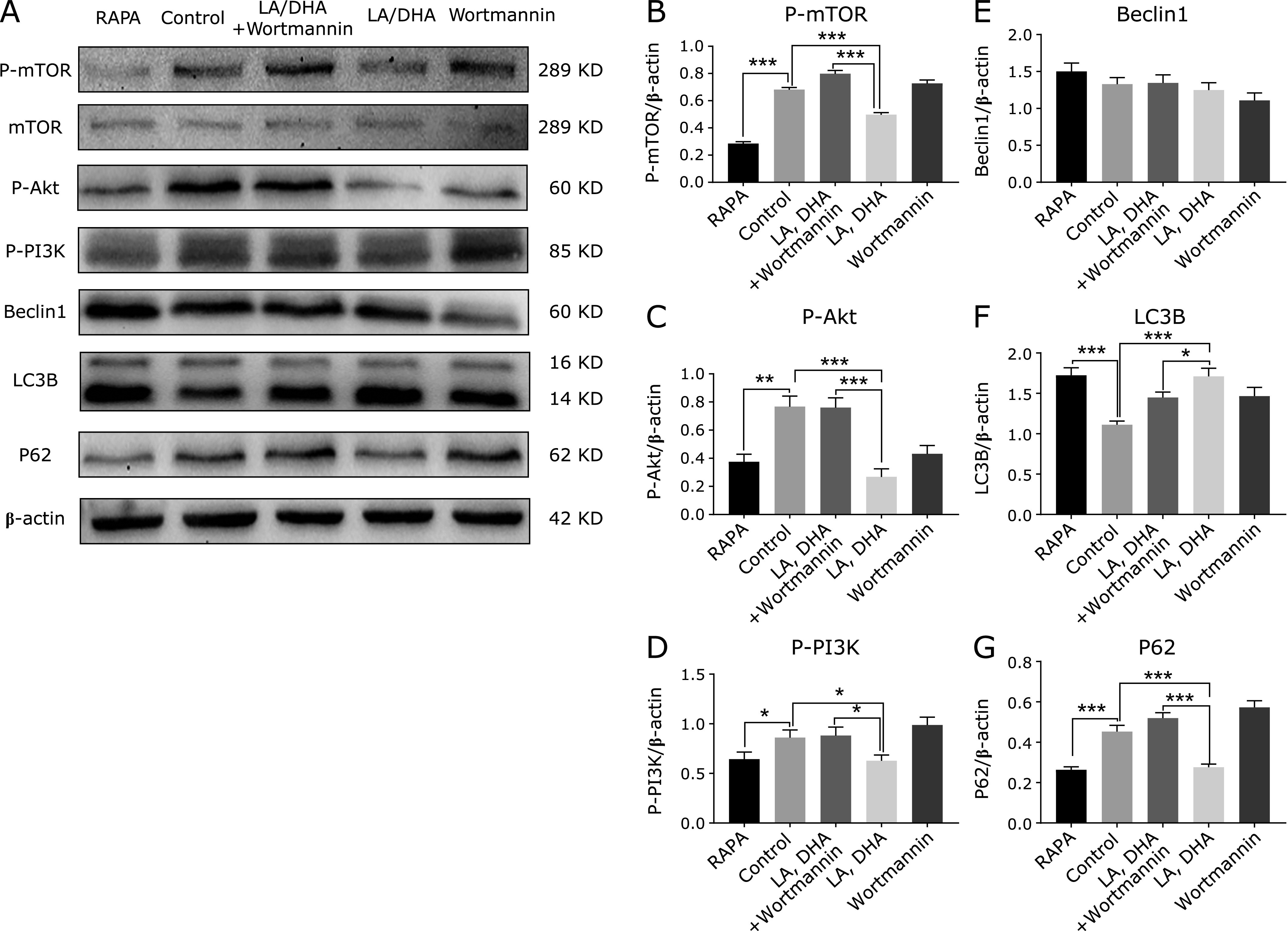
LA/DHA induce Raw264.7 cells autophagy through the PI3K-Akt-mTOR signaling pathway. (B)–(G) respectively show the expression of P-mTOR, P-Akt, P-PI3K, BECN1, LC3B and P62 proteins in each treatment group. The Western blot results of autophagy mechanism in (A)–(G) showed that the expression levels of P-mTOR, P-Akt and P-PI3K proteins in the LA/DHA group were all decreased, LC3B protein levels were significantly increased (*p<0.05, **p<0.001, ***p<0.0001), while there was no significant difference in BECN1 protein (*p*>0.05).

Results are shown in Fig. 4, LA/DHA was added to cells at a concentration of 100 µmol/L in a ratio of 6:1 for 24 h as LA/DHA group. Compared with the control group, the expression of LC3B in the LA/DHA group increased, and the P62, P-mTOR, P-Akt and P-PI3K all decreased with the difference being statistically significant (*p*<0.05). However, there was no significant difference in BECN 1 protein expression compared with the control group, and the difference was not statistically significant (*p*>0.05).

### Extracellular bacteria count

**Fig. 5:**
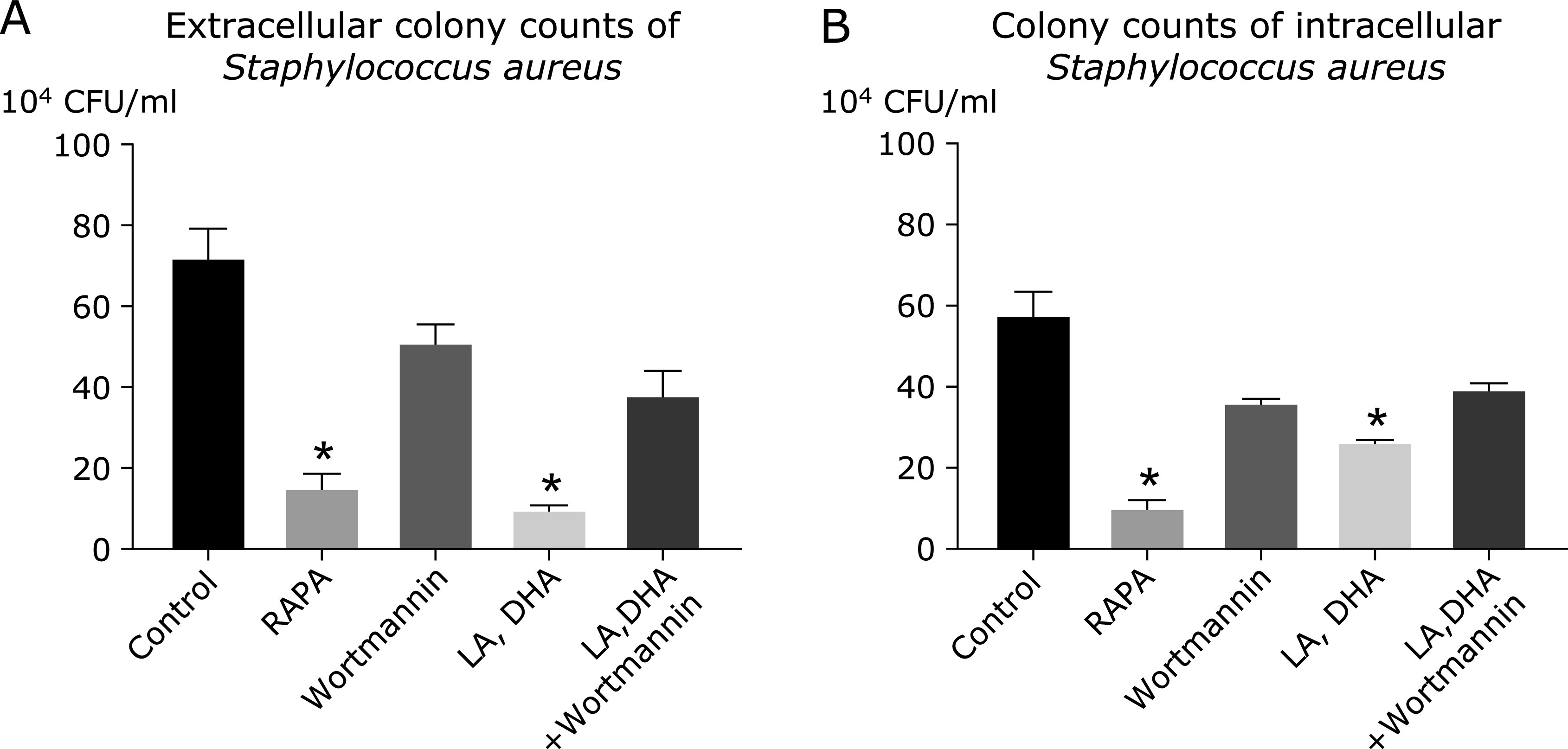
The count results of extracellular and intracellular bacteria. The extracellular colony count results of undevoured *Staphylococcus aureus* in (A) showed that the total number of colonies in the control group was 7.2 × 10^5^ CFU/ml, 1.5 × 10^5^ CFU/ml in the Rapamycin group, 5.1 × 10^5^ CFU/ml in the Wortmannin group, 1.0 × 10^5^ CFU/ml in the LA/DHA (6:1, 100 µmol/L) group, and 3.8 × 10^5^ CFU/ml in the Wortmannin group [LA/DHA (6:1, 100 µmol/L) + Wortmannin] group. The intracellular colony count results in (B) showed that the total number of colonies in the control group was 5.8 × 10^5^ CFU/ml, 1.0 × 10^5^ CFU/ml in the Rapamycin group, 3.6 × 10^5^ CFU/ml in the Wortmannin group, 2.63 × 10^5^ CFU/ml in the LA/DHA (6:1, 100 µmol/L) group, and 3.93 × 10^5^ CFU/ml in the Wortmannin intervention group [LA/DHA (6:1, 100 µmol/L) + Wortmannin] group (*p<0.0001).

**Fig. 6:**
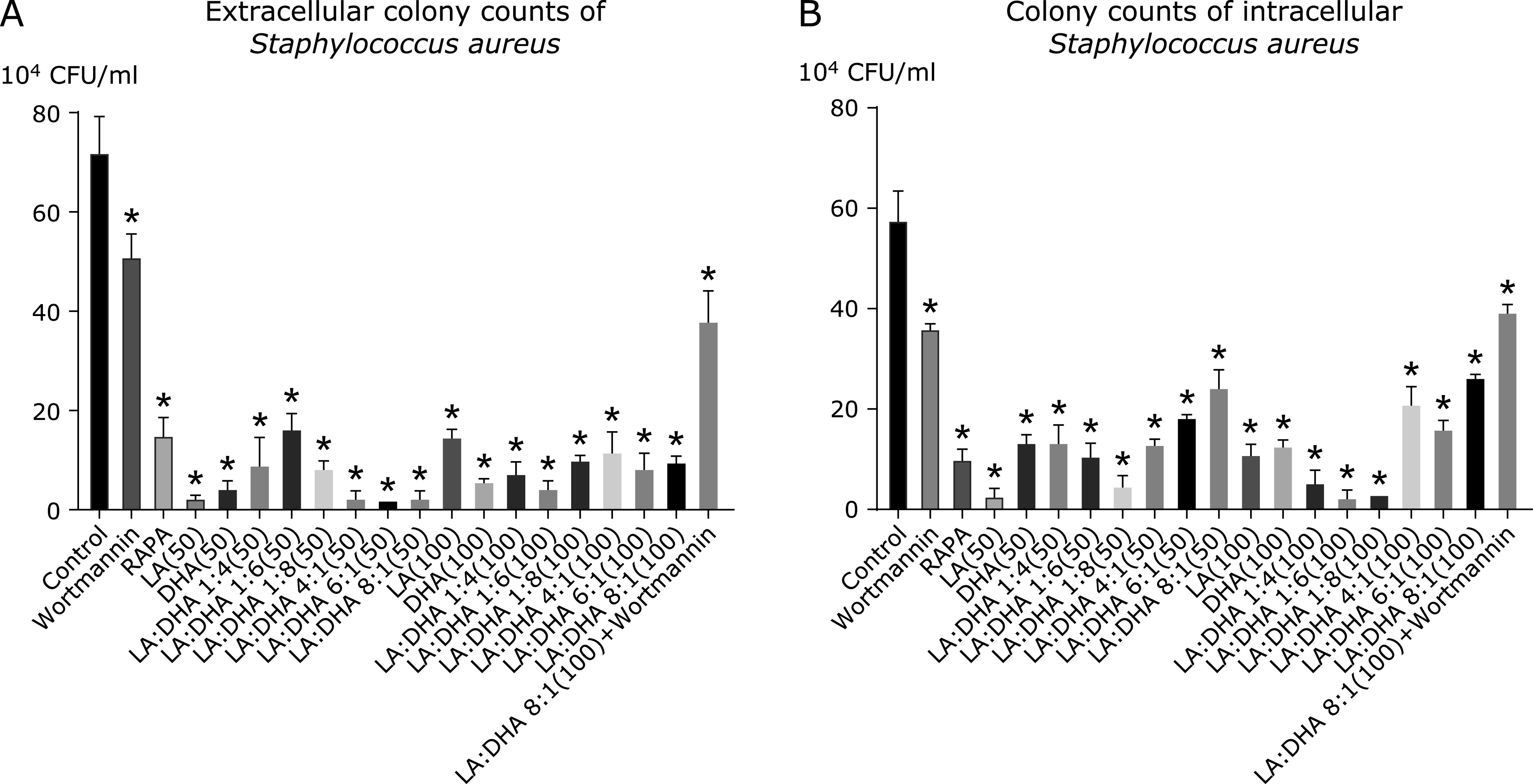
Various dose and concentration groups treated with LA and DHA could improve the phagocytosis ability of macrophages to extracellular bacteria and enhance the damage of macrophages to intracellular bacteria to some extent to a certain extent. (A) and (B) respectively showed the count results of extracellular and intracellular bacteria. The extracellular colony count results of uncleared *Staphylococcus aureus* in (A) showed that the number of uncleared *Staphylococcus aureus* in the control group was significantly higher than that in the various dose and concentration groups treated with LA and DHA. The intracellular colony count results in (B) showed that the amount of residual *Staphylococcus aureus* in macrophages in the control group was much higher than that in the different proportions and concentrations of LA and DHA groups (*p<0.05).

We used the strong autophagy induced by LA/DHA for 24 h at the concentration of 100 µmol/L, and the ratio of 6:1 as LA/DHA group. Counting results showed that the total number of colonies in the control group was 7.2 × 10^5^ CFU/ml, 1.5 × 10^5^ CFU/ml in the Rapamycin group, 5.1 × 10^5^ CFU/ml in the Wortmannin group, 1.0 × 10^5^ CFU/ml in the LA/DHA (6:1, 100 µmol/L) group, and 3.8 × 10^5^ CFU/ml in the Wortmannin intervention group [LA/DHA (6:1, 100 µmol/L) + Wortmannin]. Compared with the control group, the number of extracellular bacteria in the Rapamycin group and LA/DHA group significantly reduced (*p*<0.05), which suggested that the ability of macrophages to phagocytosis of pathogenic bacteria enhanced (Fig. 5). To further explore whether this effect has a dose-dependent effect, we treated Raw264.7 cells with different doses and concentrations of LA and DHA for 24 h. The extracellular colony count results of uncleared *Staphylococcus aureus* in A (Fig. 6) showed that the number of uncleared *Staphylococcus aureus* in the control group was significantly higher than that in the various dose and concentration groups treated with LA and DHA (*p*<0.05).

### Intracellular bacteria count

The total number of CFU in the control group was 5.8 × 10^5^ CFU/ml, that in the Rapamycin group was 1.0 × 10^5^ CFU/ml, that in the LA/DHA group was 2.63 × 10^5^ CFU/ml, that in the Wortmannin group was 3.6 × 10^5^ CFU/ml, and that in the Wortmannin intervention group [LA/DHA (6:1, 100 µmol/L) + Wortmannin] was 3.93 × 10^5^ CFU/ml (Fig. 5). Compared with the control group, the number of intracellular bacteria in the Rapamycin group and LA/DHA group significantly reduced (*p*<0.05). As in the latter half of the bacterial phagocytosis test, we also treated Raw264.7 cells with different doses and concentrations of LA and DHA for 24 h. The intracellular colony count results in B (Fig. 6) showed that the amount of residual *Staphylococcus aureus* in macrophages in the control group was higher than these in the different proportions and concentrations of LA and DHA groups (*p*<0.05).

## Discussion

The results of Cell Counting Kit-8 assay showed that LA/DHA had no promoting and toxicity or inhibition effect on Raw264.7 cells. Therefore, the effect and interference of cell death caused by toxicity of LA/DHA on autophagy can be excluded. Our studies have shown LA/DHA could induce autophagy of Raw264.7 cell, the strong effect on autophagy by the concentration is 100 µmol/L, the ratio is 6:1 of LA/DHA, and the treatment time is 24 h. And we found that under the same concentration and ratio treatment of LA and DHA, the expression level of LC3B vary from treatment time. Unlike the control group, the LA/DHA group presented red and yellow fluorescent spots after merging, which was caused by the quenching of green fluorescent spots in an acidic environment after the fusion of autophagosome and lysosome. The treatment of LA, DHA in a ratio of 6:1 at a concentration of 100 µmol/L for 24 h significantly lead to a substantial number of autophagosomes (yellow) as well as autolysosomes (red), indicating that the treatment with LA and DHA could enhance autophagy flux and the autophagy flux was smooth. The autophagy mechanism from the Western blot, the expression levels of P-mTOR, P-Akt and P-PI3K proteins in the LA/DHA group were all decreased, LC3B protein levels were significantly increased, and there was no significant difference in BECN 1 protein. But when the autophagy inhibitor Wortmannin and LA/DHA (6:1, 100 µmol/L) were used together to interfere with Raw264.7 cells, we got the opposite results suggesting that LA, DHA could induce Raw264.7 cells autophagy through the PI3K-Akt-mTOR signaling pathway. Compared with the control group, the number of intracellular and extracellulars aureus colonies in the LA/DHA group was significantly reduced, indicating that the autophagy induced by LA/DHA can enhance the phagocytosis and killing ability of macrophages to intracellular parasitic bacteria.

At present, the recommended value of *n*-6/*n*-3 PUFAs were vary from country to country. Canada recommends the ratio was 4–10:1, and the United Nations Food and Agriculture Organization was 5–10:1, the ratio of *n*-6/*n*-3 PUFAs recommended by Chinese Nutrition Society was 4–6:1, Japan was 4:l, and USA was 2.3:l. However, the reasonable ratio of the *n*-6/*n*-3 PUFAs is still in dispute.^(23)^ When we intake *n*-6/*n*-3 PUFAs, different biological effects will occur through the transformation and metabolism in the body, therefore, the number of *n*-6/*n*-3 PUFAs intake and the optimal ratio of the two are very important.^(24)^ In our study the strong effect on autophagy by the concentration is 100 µmol/L, the ratio is 6:1 of LA/DHA, and the treatment time is 24 h, it may be helpful for appropriate supplement of *n*-6/*n*-3 PUFA.

Evidence suggests that ω-3 PUFAs enhance autophagy activity and protect against acute immune overreaction due to severe liver damage.^(7)^ In recent years, DHA and EPA have received the most reports on autophagy, DHA was used to treat BMDMs cells and was found to promote autophagosome formation of macrophages.^(25)^ This conclusion is consistent with the conclusions obtained by Caviglia *et al.*^(26)^ from McArdle RH7777 cell. In addition, DHA can also induce autophagy of liver T cells under the stimulation of concanavalin A.^(7)^ On the other hand, studies have shown that DHA and EPA can not only inhibit the proliferation of A549, but also induce the autophagy of A549 in a dose-dependent and time-dependent.^(27)^ DHEA and EPEA, the two ethanolamide derivatives of DHA and EPA, also induce autophagy in breast cancer cells.^(28)^

In recent years, many scholars pay attention to the effect of ω-6 and ω-3 PUFAs on autophagy. DHA can induce complete autophagy of multiple myeloma, peripheral blood monocytes and dendritic cells without affecting their viability.^(29)^ When we measured the autophagy flux of peritoneal macrophages, monocytes or liver cell in mice, we found that diets rich in ω-6 and ω-3 PUFAs increased the level of LC3-II in aorta and liver.^(30)^ It suggested that the autophagy activity was enhanced. Study on prostate cancer cells showed that the level of LC3-II was increased in PC 3 and DU 145 cells after treated with DHA, when PC 3 and DU 145 cells were exposed to high concentrations of DHA, phosphorylation levels of Akt and mTOR decreased, but total Akt and mTOR levels showed no significant changes, it means that inhibition of Akt-mTOR pathway activates autophagy induce by DHA.^(9)^ Our results are consistent with relevant reports, LA/DHA could increase the level of LC3B protein and decrease P62 protein, and strengthen autophagy flux.

At present, it has been proved that *Staphylococcus aureus* can survive in vacuoles for a moment after being eaten by phagocytes, and then dissolve host cells and escape.^(31)^ *Staphylococcus aureus* invade macrophages and activates autophagy.^(21)^ As the innate defense system of the body, autophagy mechanism can engulf and kill microorganisms to resist various types of pathogens by phagocytes, playing a key role in host defense. Only a small group of pathogens can survive in these cells, thus avoiding host defense.^(32)^ Lv *et al.*^(21)^ found that autophagy induced by *Staphylococcus aureus* enhanced the phagocytic capacity of macrophages, but some studies suggested that *Staphylococcus aureus* used VraSR regulatory system to block autophagy flux, so as to improve the intracellular survival rate.^(17)^ Some scholars believe that autophagy induction may contribute to the intracellular survival of pathogenic bacteria and may be harmful to the host,^(18,31–33)^ while others hold the opposite view.^(17,34)^ Therefore, the relationship between autophagy and *Staphylococcus aureus* infection remains to be studied.

In summary, we demonstrate, for the first time, that LA/DHA could induce autophagy through PI3K-Akt-mTOR pathway. Autophagy induced by LA/DHA can help cells to devour intracellular and extracellular *Staphylococcus aureus*. This study provides a new insight of the LA/DHA autophagy induction in Raw264.7 cells against *Staphylococcus aureus*.

## Author Contributions

The authors’ responsibilities were as follows—LYX performed the Western blot experiments, analyzed the results, and drafted the manuscript. MLW, JCL, XY, YJZ, QL, JW and DH helped Western blot experiments, analyzed the results. XRT and MM contributed to the ideas presented in this article; MM contributed to the experimental concepts and design, provided scientific direction, and finalized the manuscript; and all authors read and approved the final manuscript.
